# Editorial: Epigenetic Regulation of Innate Immunity

**DOI:** 10.3389/fimmu.2021.713758

**Published:** 2021-06-17

**Authors:** Menno P. J. de Winther, Tanapat Palaga

**Affiliations:** ^1^ Experimental Vascular Biology, Department of Medical Biochemistry, Amsterdam Cardiovascular Sciences, Amsterdam Infection and Immunity, Amsterdam University Medical Centers, Amsterdam, Netherlands; ^2^ Department of Microbiology, Faculty of Science and Center of Excellence in Immunology and Immune-mediated Diseases, Chulalongkorn University, Bangkok, Thailand

**Keywords:** epigenetics, innate immunity, histone modifications, long noncoding RNA (lncRNA), diseases

Innate immune cells form a first line of defense against invading pathogens. It comprises a variety of cells including neutrophils, natural killer cells (NK), and macrophages and their precursors monocytes. They are important in host defense for killing of pathogens, regulating immune cell recruitment and activation but also serve trophic functions, maintain tissue homeostasis and control resolution phases of inflammatory responses. In the context of health and disease, innate immune cells display a wide variety of functional phenotypes beyond the old dogma of M1 and M2 macrophages. It is now becoming clear, driven by innovative technologies such as mass cytometry or single cell sequencing, that tissue microenvironments drive very specific phenotypes in relation to disease and this is revolutionizing our understanding of pathology that will provide new opportunity for future personalized interventions.

Inflammatory processes and innate immune cells are tightly regulated by epigenetic mechanisms. These processes include genomic DNA methylation and modification of histones by methylation or acetylation, all of which control the epigenetic landscape, chromatin accessibility and thereby the function of immune cells in disease (1). Moreover, RNA modulatory mechanisms such as long non-coding RNAs (lncRNAs) or chemical modifications of RNA molecules also impact on the transcriptional programs. Epigenetic processes control cellular differentiation event and also direct responses of innate immune cells to systemic and local inflammatory triggers (2). Importantly, enzymes that control the epigenetic machinery are often amenable to pharmacological modulation and their targeting represents a rapidly evolving therapeutic space. Pharmacological blockade of signaling pathways that impact on epigenetic processes also provides therapeutic options for treatment of disease (3). Because epigenetic regulation conditions cells to respond to specific external and internal cues with more accuracy, targeting epigenetics for therapy is expected to be more effective with longer duration of impact.

In this Research Topic on epigenetic regulation of innate immunity, three reviews focus on the involvement of epigenetics in liver resident Kupffer cells, sex hormones and epigenetics in innate immune responses and RNA modifying enzymes on immunity. Four research articles reported on the roles of epigenetic modifying enzymes (HDAC, Ezh2) in macrophages, epigenetic mark readers BRD2/4 in NK and circular RNA in innate immune cells.

Bennett et al. summarize an up-to-date view on epigenetics in development and differentiation of Kupffer cells (KC) and the epigenetic changes accompanying non-alcoholic fatty liver disease (NAFLD) and non-alcoholic steatohepatitis (NASH). Using transcriptomic and transcription factor (TF) binding analysis, the key regulators of KC differentiation are Notch signaling, SpiC and SMAD in collaboration with lineage determining TFs including PU1 and CEBP. Notably, changes in histone modifications (H3K27) and lncRNAs were observed in KC during the transition to NASH. These changes culminate in the reprograming of liver X receptor (LXR)α that affect its recruitment to target promoters.

Epigenetics that are under the influence of sex hormones are proposed to affect sexual dimorphism in innate immunity. Shepherd et al. reviews the molecular reprograming of innate immunity by sex hormones, resulting in differential responses to infections between male and female. Sex hormones through binding to nuclear hormone receptors recruits co-activators, co-repressors, and epigenetic modifying enzymes to the promoters of its target genes. The most obvious impacts are discussed during hormonal changes such as pregnancy and menopause in female. The influences of sex hormones on epigenomes of innate immune cells may also involve inflammation in aging population (inflammaging).

The importance of RNA N^6^-methyltransferase (m6A) is reviewed by Tang et al. This modification affects RNA stability, degradation, the ability to be recognized as PAMPs. Writers (METTL14/METTL3), erasers (ALKBH5/FTO) and readers (such as FMRP) of m6A perform the modification in nucleus and cytoplasm which plays a critical role in DC maturation and thus affects adaptive immune responses in autoimmune and cancer. Examples for viruses that may hijack this RNA modifier to camouflage from host recognition are given, and the authors argue that targeting m6A should be exploited to enhance anti-viral and anti-tumor immunity.

The two research articles investigate histone modifying enzymes in responses of monocytes/macrophages and its potential impact on inflammatory-driven disease. Neele et al. investigate the role of Ezh2, a subunit of polycomb repressive complex (PRC) in myeloid cells, especially macrophages and neutrophils. Ezh2 catalyzes methylation of H3K27 that results in repressive chromatin. Deficiency of Ezh2 in myeloid linage cells reduced atherosclerosis development and inflammatory foam cells while impairing neutrophil migration. In a separate study, Ghiboub et al. reported the involvement of histone deacetylases (HDACs) in human monocytes and macrophages. The authors reported that HDAC3 but not HDAC6 regulates responses of monocytes/macrophages to M1/M2 promoting stimuli and repeated LPS stimulation. Both works highlighted the potential targeting epigenetic modifying enzymes for inflammatory-related conditions.

Cribbs et al. focus on the roles of BET bromodomain containing protein (BRD) on NK cell functions. BRDs recognize acetylated lysine residues on histones and acts as scaffold protein for recruitment of TF or protein complex. Using specific inhibitors for BRD2 and 4, they report that BRD2 regulates its inflammatory and cytolytic activity. Using scRNA-seq and ChIP-seq, the authors further reveal the heterogeneity of NK responses to BET bromodomain inhibition that leads to reduced promoter occupancy of BRD2/4. The results from this study warrant further investigation on the potential use NK in cancer immunotherapy.

Yang et al. focus on a circular non-coding RNA (circRNA), that acts as sponge against specific miRNA and reduce its availability. Observations of upregulation of Hsa_circ_0060450 in monocytes of type 1 DM patients led the authors to investigate this circRNA. They observed that miR-199a-5p is the target of Hsa_circ_0060450. Because miR-199a-5p promotes type I IFN responses by targeting phosphatase SHP2, increasing Hsa_circ_0060450 may lead to reduced type I IFN responses in type 1 DM. Therapy targeting this cicrRNA may be beneficial for type 1 DM patients in the context of viral infection.

Future investigations of components in different cell types under different epigenetic contexts may aid in developing more specific drug(s) that can be used to treat diseases of innate immune dysregulations.

## Author Contributions

MW and TP equally contributed in preparing this Editorial. All authors contributed to the article and approved the submitted version.

## Conflict of Interest

The authors declare that the research was conducted in the absence of any commercial or financial relationships that could be construed as a potential conflict of interest.
